# Bone Bruise of the Thoracic Spine Caused by Mild Physical Activity in Children

**DOI:** 10.1155/2017/8451797

**Published:** 2017-11-28

**Authors:** Kenji Yokoyama, Kenji Endo, Yoichiro Takata, Fumitake Tezuka, Hiroaki Manabe, Kazuta Yamashita, Toshinori Sakai, Takashi Chikawa, Akihiro Nagamachi, Koichi Sairyo

**Affiliations:** ^1^Department of Orthopedics, Tokushima University, Tokushima, Japan; ^2^Endo Orthopedic Clinic, Tokushima, Japan

## Abstract

Vertebral bone bruise (VBB) in children commonly occurs following a fall from a height, and more than one vertebral body may be affected. We encountered 6 children each with a single VBB caused by mild physical activity. All the children had tenderness on the corresponding spinous process with no neurologic findings. Magnetic resonance imaging (MRI) showed typical findings of VBB in all cases. The children were treated conservatively with a soft thoracolumbar brace and instructed to rest with no physical activity for a month. At follow-up 1 month later, the back pain had diminished, and the signal changes seen on MRI had disappeared in all cases. We conclude that mild physical activity may be a cause of VBB in children and good clinical results can be achieved by using a soft thoracolumbar brace and rest.

## 1. Introduction

Spinal injury is relatively rare in children, with a reported incidence of 0.6% [1, 2]. Falls and motor vehicle accidents account for more than 75% of injuries to the thoracolumbar spine in children [3, 4]. To date, there have been no reports of spinal injury caused by mild physical activity in this age group.

A bone bruise is defined as a bone marrow lesion with microtrabecular fracture of cancellous bone, without associated cortical bone fracture, and is often observed at the knee and the ankle. Magnetic resonance imaging (MRI) is the preferred imaging modality and the first choice for diagnosis of a bone bruise.

A vertebral bone bruise (VBB) is defined as band-like zones of high signal intensity on T2-weighted imaging (T2WI) and decreased signal intensity on T1-weighted imaging (T1WI), without associated fracture or deformation of the endplate [5]. The main cause of a VBB is an axial compressive load to the anterior column of the spine. In this case series, we demonstrate that mild physical activity can cause VBB in children.

## 2. Case Presentation

The authors have obtained the patients' parent's informed written consent to publish this case report.

**Figure 1:**
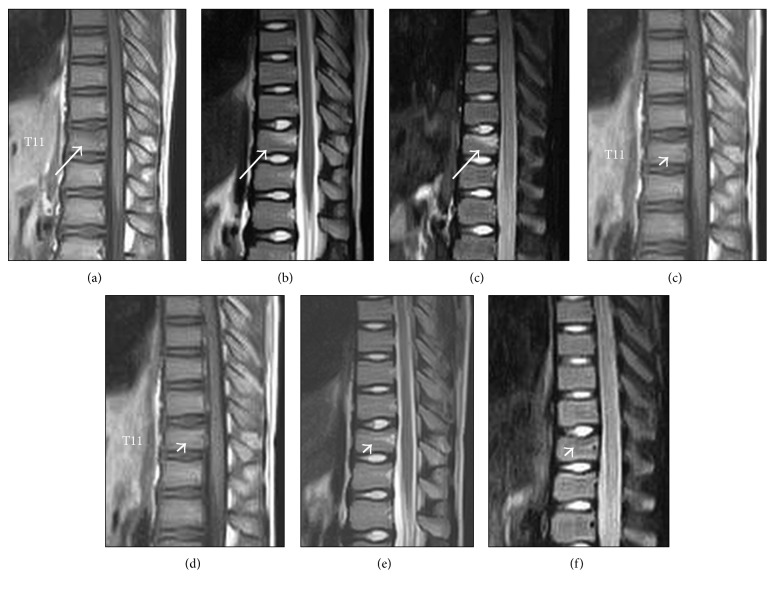
Magnetic resonance images at the initial visit showing low signal on T1WI (a), relatively high signal on T2WI (b), and high signal on short tau inversion recovery (STIR) image (c) in the upper part of the vertebral body at T11 without deformation of the cortical endplate (arrow). At 1-month follow-up later, signal changes had diminished on T1, T2, and STIR images, respectively (d, e, and f, arrowhead).

A 10-year-old boy (Case 3) presented with back pain after performing a handstand without a fall during a physical education session. He had no significant past medical history. At the initial visit, there was tenderness on his back, especially on the spinous process at T11. Neurologic findings were normal. Plain radiographs were unremarkable. MRI scans indicated low signal on T1WI, relatively high signal on T2WI, and high signal on short tau inversion recovery (STIR) images in the upper part of the vertebral body at T11 without deformation of the cortical endplate (Figure 1). There was no high signal in the posterior elements of the spine, such as the interspinous ligament. A diagnosis of VBB was made. The patient was treated conservatively with a soft thoracolumbar brace and instructed not to participate in physical activity for a month. At follow-up 1 month later, the patient had no back pain, the tenderness on the spinous process had diminished, and the signal changes in the vertebral body seen on T1WI, T2WI, and STIR images had disappeared (Figure 1). There was no residual deformation of the cortical endplate.

The other 5 children had similar clinical manifestations (Table 1). The onset of back pain in all 6 children coincided with mild physical activity, such as performing a handstand, jumping rope, or Japanese box vaulting. None of the children reported a fall. We did not check any blood test for all 6 patients because they had no past medical history. There was tenderness on the corresponding spinous process at the initial visit in all cases. Imaging findings were similar; plain radiographs were unremarkable, and MRI scans showed low signal on T1WI and high signal on T2WI and STIR. In all cases, the back pain and signal changes seen on MRI resolved after conservative treatment with a brace and rest for 1 month.

## 3. Discussion

The diagnosis of a bone bruise is made on MRI, and the term “bone contusion” is used synonymously. Bone bruise was first described as an entity by Mink and Deutsch in 1989 [6]. The characteristic MRI findings are low signal intensity on T1WI and high signal intensity on liquid-weighted sequences. Symptoms and associated radiographic findings typically resolve after a few months, and the clinical outcome is good.

Teli et al. identified 30 VBBs in 285 nonfractured thoracic and lumbar vertebrae in adults, associated with 21 fractures of other vertebrae. They also noted that VBB within a thoracic or lumbar vertebral body did not cause significant vertebral wedging in adults [7].

The low incidence of spinal column injuries in children is mainly attributed to the anatomic characteristics of the axial skeleton in childhood. The main traumatic causes of spinal injury in children are accidents in the home and road traffic accidents. With increasing age, sporting and recreational injuries have become increasingly common [8].

Scheunemann et al. reported a case series of 66 children with VBB diagnosed on MRI in the German literature [9]. The main cause of injury in those children was a fall from a height (over 1 m), such as down a flight of stairs or from a horse. Typically, more than one vertebral body around the thoracolumbar junction was affected (range 1–8; 3.05 on average).

In contrast, all 6 children in our study had pain after very mild physical activity rather than a fall from a height, and a single spinal level was affected in all cases. However, the mechanism of VBB might be similar to that in the German report. Axial force to the anterior spinal column seems to be the main cause of VBB. The difference is the amount of mechanical force exerted on the vertebral body. In the German report, the force was higher than in our cases. To the best of our knowledge, there are no reports of VBB due to mild physical activity in children.

The advantage of this report is that MRI scans were available for both the initial visit and the follow-up visit 1 month later. In all patients, signal changes in the vertebral body at the initial visit had diminished at follow-up, and the back pain had also disappeared at this time. Therefore, the cause of back pain in these patients was VBB, and treatment with a brace and rest for a month was successful. Interestingly, all 6 children were living in a small town with a population of 20,000, and all 6 VBBs occurred in 2 years. It might be more common than we expected.

In conclusion, mild physical activity may cause VBB in children. MRI is a good diagnostic modality because the affected vertebral body shows signal changes. A soft thoracolumbar brace and rest can achieve good clinical results.

## Figures and Tables

**Table 1:** Demographic characteristics and history of injury in 6 children with vertebral bone bruise.

Case	Age (years)	Sex	Physical activity	Level affected
1	9	Girl	Jumping rope	T5
2	9	Boy	Jumping rope	T6
3	10	Boy	Handstand	T11
4	11	Boy	Jumping rope	T7
5	12	Boy	Physical education	T4
6	13	Boy	Japanese box vaulting	T6
